# Protocol for a feasibility study of a cohort embedded randomised controlled trial comparing **NE**phron **S**paring **T**reatment (NEST) for small renal masses

**DOI:** 10.1136/bmjopen-2019-030965

**Published:** 2019-06-11

**Authors:** Joana B Neves, David Cullen, Lee Grant, Miles Walkden, Steve Bandula, Prasad Patki, Ravi Barod, Faiz Mumtaz, Michael Aitchison, Elena Pizzo, Veronica Ranieri, Norman Williams, William Wildgoose, Kurinchi Gurusamy, Mark Emberton, Axel Bex, Maxine G B Tran

**Affiliations:** 1 Department of Surgical Biotechnology, Division of Surgery and Interventional Science, University College London, London, UK; 2 Specialist Centre for Kidney Cancer, Royal Free London NHS Foundation Trust, London, UK; 3 Department of Radiology, Royal Free London NHS Foundation Trust, London, UK; 4 Department of Interventional Radiology, University College London Hospitals NHS Foundation Trust, London, UK; 5 Department of Medicine, Centre for Medical Imaging, University College London, London, UK; 6 Department of Urology, Barts Health NHS Trust, London, UK; 7 Department of Applied Health Research, Institute of Epidemiology and Health, University College London, London, UK; 8 Tavistock and Portman NHS Foundation Trust, London, UK; 9 Patient Representative, London, UK; 10 Department of Surgery, Faculty of Medical Sciences, University College London, London, UK; 11 Department of Urology, University College London Hospitals NHS Foundation Trust, London, UK

**Keywords:** kidney tumours, cohort embedded randomised controlled trial, small renal mass, cryoablation, partial nephrectomy

## Abstract

**Introduction:**

Small renal masses (SRMs; ≤4 cm) account for two-thirds of new diagnoses of kidney cancer, the majority of which are incidental findings. The natural history of the SRM seems largely indolent. There is an increasing concern regarding surgical overtreatment and the associated health burden in terms of morbidity and economy. Observational data support the safety and efficacy of percutaneous cryoablation but there is an unmet need for high-quality evidence on non-surgical management options and a head-to-head comparison with standard of care is lacking. Historical interventional trial recruitment difficulties demand novel study conduct approaches. We aim to assess if a novel trial design, the cohort embedded randomised controlled trial (RCT), will enable carrying out such a comparison.

**Methods and analysis:**

Single-centre prospective cohort study of adults diagnosed with SRM (n=200) with an open label embedded interventional RCT comparing nephron sparing interventions. Cohort participants will be managed at patient and clinicians’ discretion and agree with longitudinal clinical data and biological sample collection, with invitation for trial interventions and participation in comparator control groups. Cohort participants with biopsy-proven renal cell carcinoma eligible for both percutaneous cryoablation and partial nephrectomy will be randomly selected (1:1) and invited to consider percutaneous cryoablation (n=25). The comparator group will be robotic partial nephrectomy (n=25). The primary outcome of this feasibility study is participant recruitment. Qualitative research techniques will assess barriers and recruitment improvement opportunities. Secondary outcomes are participant trial retention, health-related quality of life, treatment complications, blood transfusion rate, intensive care unit admission and renal replacement requirement rates, length of hospital stay, time to return to pre-treatment activities, number of work days lost, and health technologies costs.

**Ethics and dissemination:**

Ethical approval has been granted (UK HRA REC 19/EM/0004). Study outputs will be presented and published.

**Trial registration:**

ISRCTN18156881; Pre-results.

**Strengths and limitations of this study**

- The main strength of this study is the use of a novel and pragmatic trial design, the cohort embedded randomised controlled trial, to improve participant recruitment.
- Trial recruitment difficulties have hindered the acquisition of level one evidence on interventional management of small renal masses (SRMs), so the study will also incorporate qualitative research techniques to assess barriers and recruitment improvement opportunities.
- Possible study limitations include the lack of generalisation of results to individuals with large renal masses or advanced disease; the open label nature of the trial’s interventional comparison and the feasibility study being single site.
- If the primary outcome (successful recruitment) is met, this will enable the implementation of a larger-scale multicentric cohort embedded randomised trial to compare percutaneous cryoablation to robotic partial nephrectomy as a management strategy for SRMs.

## Introduction

Over 12 000 patients are diagnosed with renal cancer in the UK every year,^1^ and the incidence is increasing by 2% annually.^2^ The number of kidney cancer cases has more than doubled since the 1970 s^1^. Small renal masses (SRMs; T1a lesions on Tumour Node Metastases (TNM)staging; ≤4 cm of largest axis) account for two-thirds of new diagnoses of kidney cancers,^3^ the majority of which are incidental findings on investigations for other ailments or non-specific symptoms.^4^

The natural history of the SRM seems largely indolent. It is known that some (up to 40%) do not grow, and the majority that do increase in size, tend to enlarge slowly, between 1 and 15 mm per year, and providing that they do not breach the 4 cm size threshold, pose a very low risk of metastasis (1%).^5 6^ Treatment options for SRMs include active surveillance, ablation (either cryoablation or radiofrequency ablation) or surgical excision (partial nephrectomy or radical nephrectomy).^7^

The mainstay in treatment of a SRM is partial nephrectomy.^7^ Partial nephrectomy is recommended whenever technically possible as it preserves kidney function while providing good long-term oncological control. However, it is complex surgery and is associated with a higher major complication rate (4.9%), compared with radical nephrectomy (1.3%).^8^ In most cases, partial nephrectomy is performed using the da Vinci surgical robot system which costs around £1.5–2 million to purchase. Robot-assisted partial nephrectomy is estimated to cost between £7000 and 11 900 per case once the purchase and maintenance of the robot are taken into account.^9^

Considering the slow growth and indolent nature of the majority of SRMs, there is an increasing concern regarding overtreatment with surgery and the associated health burden both in terms of morbidity and economy. An alternative treatment option that also preserves renal function for SRMs is cryoablation which is now almost exclusively performed percutaneously under CT guidance. Cryoablation uses argon probes to freeze and kill the tumour, and is performed under a short general anaesthetic, usually with a single night admission. Present guideline recommendations for clinical practice are based on low-level evidence (level of evidence is 3 based on the Oxford Centre for evidence-based medicine system) and advocate cryoablation for elderly patients, or those with significant comorbidities due to the increased morbidity associated with surgical excision.^7^ Cryoablation is also an attractive option for patients with familial or sporadic multifocal bilateral tumours, as an alternative to multiple repeated surgical procedures and to increased renal function preservation compared with partial nephrectomy. Longer-term oncological outcomes from cryoablation are emerging and indicate equivalent oncological control at 9 years when compared with partial nephrectomy—this means that the distinction in patient selection criteria for cryoablation or for partial nephrectomy is becoming less clear.^10^ A recent large retrospective study at the Mayo Clinic (USA) reported that recurrence-free survival was similar for both partial nephrectomy and cryoablation.^11^ Overall survival was superior after partial nephrectomy, but this is likely due to selection bias as patients who underwent partial nephrectomy were significantly younger and had lower Charlson comorbidity scores.^11^

A recent systematic review of cryoablation for SRM reports on the excellent functional outcomes, rapid recovery and low rates of complications, supporting its use as an alternative minimally invasive treatment modality.^12^ Cryoablation can also be performed at a significantly lower cost than partial nephrectomy. Direct comparison studies in the UK are not yet available, but a study in Michigan, USA, reported the mean total cost for percutaneous cryotherapy to be almost half the cost of open or robot-assisted partial nephrectomy: $6067 versus $11 392 or $11 830 (p<0.0001).^13^

Previous attempts of randomised controlled trials (RCTs) in the context of SRM, for example CONSERVE (A feasibility study for a multicentre randomised controlled trial to compare surgery (partial nephrectomy) with needle ablation techniques (radiofrequency ablation/cryotherapy) for the treatment of people with small renal masses (4cm)) (comparing partial nephrectomy with radiofrequency or cryoablation; ISRCTN23852951) and SURAB (Study comparing ABlation with active SURveillance, in the management of incidentally diagnosed small renal tumours: a feasibility study) (comparing active surveillance and cryoablation; ISRCTN31161700) were not completed. This has been due to a number of factors, most notably; delays in obtaining National Health Service (NHS) permissions for multicentre sites, delays in treatment in the ablative arms due to lack of radiological capacity and insufficient trial duration (24 months for CONSERVE). Overall, CONSERVE involved four sites with a target of 60 participants and recruited 17. SURAB was stopped early due to the recruitment of only five patients when they had predicted 32 by a pre-defined milestone time point.

This means that we currently do not know how best to advise our patients other than by presenting clinical outcome variables of hospital stay and recovery. Current evidence available on the treatment outcomes of SRMs now point to a situation of clinical equipoise between partial nephrectomy and cryoablation, and well-designed clinical trials are crucial to clarify the treatment strategy, minimise overtreatment and improve overall patient outcome. This has been identified as a priority area by the renal cancer subgroup of the National Cancer Research Institute.^14^

Surgical RCTs are notoriously challenging to design and deliver. The main obstacles include professional resistance due to a perceived lack of clinical equipoise, lack of ability to ‘blind’ treatment allocation and an inherent and understandable reluctance for patients to accept treatment allocation based on chance alone. An alternative trial design for effective comparison of treatments has been reported, the ‘cohort embedded RCT’ and has been used successfully in chronic obstructive pulmonary disease and colorectal cancer trials.^15 16^ While informed consent is regathered for the intervention trial, the data from the cohort are based on prior broad consent. Unlike the traditional RCT design, the control participants are informed about prospective relevant trials within the cohort.^17^

The proposed cohort embedded RCT study design fully incorporates recruitment intervention techniques to pre-empt and identify recruitment issues early, ensuring successful delivery of both the initial feasibility trial and full trial. Qualitative research has been shown to improve recruitment in RCTs.^18^ The study design also allows the future addition of further intervention arms, that is, multiple RCTs can be embedded in the study cohort (subject to separate ethics approval). The advantages are that long- and short-term outcomes can be measured and compared, and natural history of the condition can also be studied. Important additional advantages include the fact that patients know which treatment they are agreeing to, the non-disruptive nature of the trial as patients receive care in routine healthcare settings and large numbers can be recruited. We conducted an online survey together with the charity Kidney Cancer UK to ascertain broad acceptability of the study’s design and willingness of patients to be recruited into nephron sparing treatment (NEST; unpublished data). Over a 2-week period, there were 99 respondents. Ninety per cent of patients indicated willingness to be followed up for life and 99% indicated that they would consent to being approached in the future if there was a new treatment or test. Ninety-nine per cent would consent for patient report outcome measures (PROMs)/questionnaires to be sent (maximum two per year), 98% would consent for blood and urine to be used for research and 99% would consent for tissue to be used for research. This online survey suggests strong support and need from the kidney cancer patient community for the broad concepts of the study.

The overarching objective of this feasibility study is to ascertain if a novel cohort embedded RCT design with recruitment improvement strategies can deliver a full-scale interventional trial comparing nephron sparing interventions in kidney cancer.

## Methods and analysis

This report complied with Standard Protocol Items: Recommendations for Interventional Trials guidelines^19^ (online supplementary document 1).

10.1136/bmjopen-2019-030965.supp1 Supplementary data

### Study design

**Figure 1:**
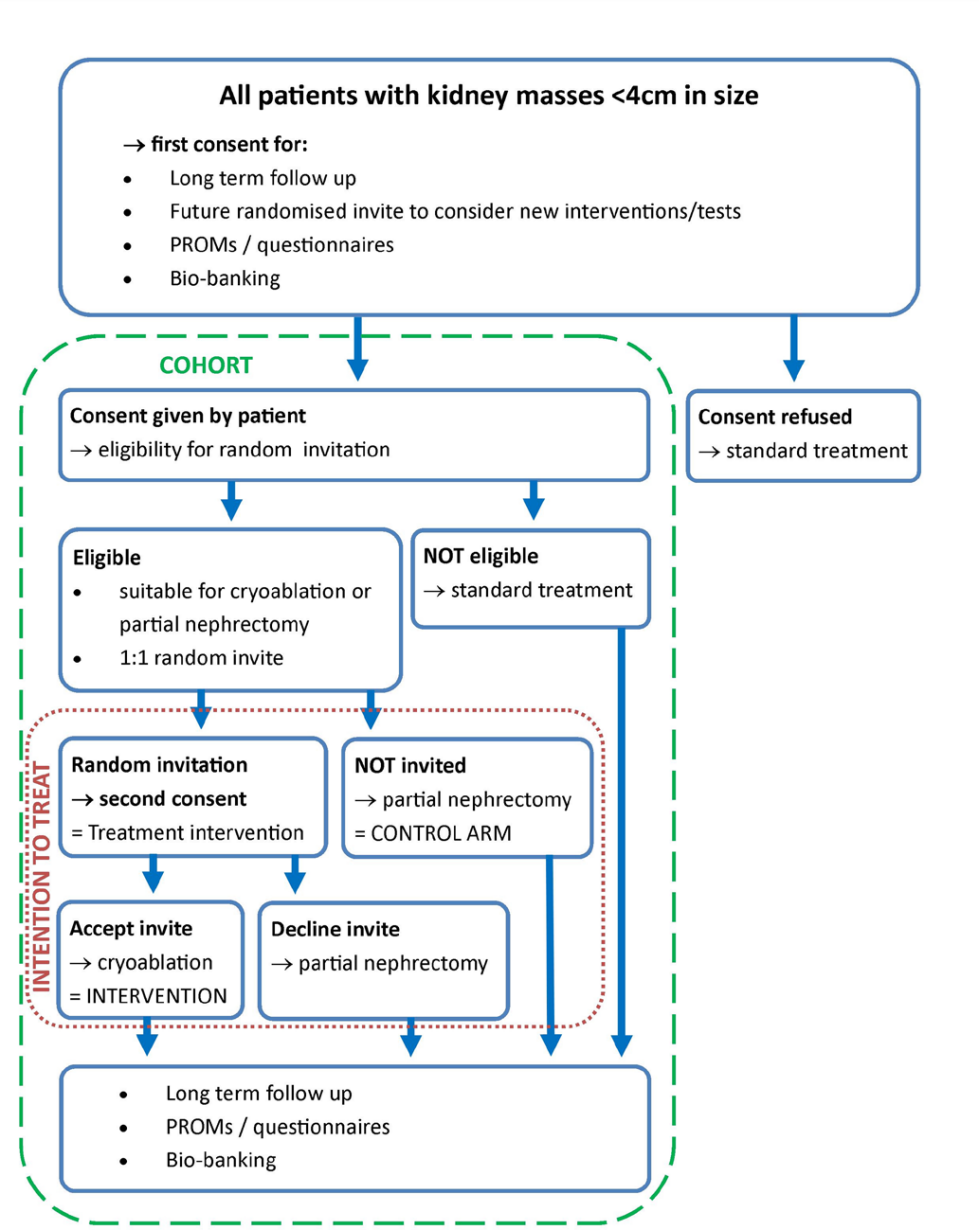
Study flow chart.

Single-centre prospective feasibility cohort study of adults that have been diagnosed with a SRM (n=200) with an open label embedded interventional RCT comparing nephron sparing techniques (figure 1 – Trial flow chart).

This pragmatic study design incorporates a two-stage consent process:

1. The first stage allows the creation of a prospective observational cohort of patients with SRMs. Cohort participants will be managed at patient and clinicians’ discretion and consent for: (a) long-term follow-up and data linkage to NHS digital and/or other central NHS registries; (b) future randomised invitation for new interventions, tests, or treatments; (c) participation in interventional trials as a control group without need of further consent; (d) PROMs/questionnaires completion; and (e) biobanking of blood/urine/tissue.
2. In the second stage, cohort participants with biopsy-proven renal cell carcinoma (RCC) who are eligible for both percutaneous cryoablation and partial nephrectomy will be randomly selected (1:1) and invited to consider percutaneous cryoablation (n=25). Participants randomly selected to consider the trial intervention (percutaneous cryoablation) will be asked for informed consent (second consent process) and will form the interventional arm. Participants not randomly selected to consider the trial intervention will receive standard of care (partial nephrectomy) and will form the control arm. By virtue of having agreed to be part of control trial arms at the first stage consent process, these participants will not require a second consent process. They will, however, be subject to surgical consent for the procedure as part of standard practice.

Patient information sheets and consents forms are available in online supplementary document 2 to 5.

### Objectives and outcomes

The primary outcome is to test the feasibility of recruitment into a cohort embedded RCT in NEST of SRMs.

Secondary outcomes are as follows:

1. Participant retention rate, measured annually by analysing the number of randomised participants retained and assessed with valid primary outcome data.
2. Health-related quality of life, measured using the previously validated EuroQol -5domains-5levels (EQ-5D-5L) questionnaire prior to treatment and 3 months following treatment
3. Treatment complications, blood transfusion, intensive care unit admission, and renal replacement requirement rates, measured using clinical records during hospital admission, at 30 days postoperative and at 6 months.
4. Length of hospital stay, time to return to pre-treatment activities and number of work days lost (in those who work), measured using clinical records and follow-up consultation (clinic or telephone) at 30 days and at 6 months.
5. Costs incurred by health technologies, measured using NHS reference costs and also private and societal costs measured using patient completed questionnaire at time of treatment, at 30 days and at 6 months.

### Study setting

The study will be conducted in a UK tertiary academic referral centre specialised in kidney cancer care.

### Eligibility criteria

The inclusion criteria for entry in cohort include adult (≥18 years of age) of any gender participants capable and who have provided informed consent and that have been diagnosed with a SRM (≤4 cm of largest axis on cross-sectional imaging). Exclusion criteria include inability to provide informed consent and advanced disease (N1 and/or M1 on TNM staging).

The inclusion criteria for the embedded RCT include, in addition to the above-mentioned criteria, biopsy-proven RCC and equal feasibility for treatment with either percutaneous cryoablation or partial nephrectomy. Exclusion criteria include any concurrent medical/surgical condition or indication, which would mean the specialist multidisciplinary team (SMDT) meeting recommends one treatment modality is more suitable than another, such as:

1. Myocardial Infarction in preceding 6 months.
2. Pulmonary disease not allowing for prolonged anaesthesia.
3. Multiple previous abdominal surgery/interventions, making surgical approach high risk.
4. Performance status ≥2.
5. Metastatic disease.
6. Charlson comorbidity index >3.
7. Patients with multifocal tumours.
8. Patients with suspected or diagnosed with inherited kidney cancer susceptibility syndromes.
9. Women that are pregnant or breast feeding (this is due to the fact that percutaneous cryoablation is done under CT guidance with associated radiation exposure).

Eligible patients will be identified at the weekly renal SMDT meeting by the chief investigator or a member of the clinical research team. All participants who wish to enter the study will be fully screened and consented by the Chief Investigator, one of the qualified clinicians involved in the study as Clinical Co-Investigator, or senior study staff when counter signed by a delegated clinician.

### Interventions

Cohort participants eligible for the embedded RCT will be randomised 1:1 and invited to consider the trial intervention (percutaneous cryoablation). Randomisation will be performed in blocks of 10 participants through the online system ’Sealed Envelope' (www.sealedenvelope.com) to ensure allocation concealment. This will be an open label trial with unblinded outcome assessment.

The control group will be composed of participants treated with standard of care (partial nephrectomy). The trial intervention will be performed by interventional radiologists under CT guidance using argon probes to freeze and kill the tumour. The standard of care will be performed by urological surgeons with da Vinci robot assistance.

No restrictions will be placed on other clinically necessary concomitant care. Follow-up for the purposes of this feasibility trial will be 6 months, but standard clinical follow-up will be at least 5 years (table 1—visit schedule and assessments). On average, patients attend two clinic appointments prior to treatment. Post-treatment follow-up is based on histological findings at biopsy/surgery but usually involves 6 monthly clinic visits for a minimum of 5 years. Participants will not be required to have any additional visits, apart from volunteers for focus groups and structured interviews.

**Table 1:** Visit schedule and assessments

Procedures	Visits								
	Screening	Baseline	Intervention	Week 4 post-intervention (only second stage of study)	Month 6 after baseline (first stage of study) or intervention (second stage of study)	Y1	Y2	Y3	Y5
Demographics		X							
Medical history		X							
Physical examination		X		X					
Consent		X	X						
Blood test		X		X	X	X	X	X	X
Imaging	X				X	X	X	X	X
Questionnaire		X		X	X	X	X	X	X
Cryoablation or partial nephrectomy			X						
Case report form		X	X	X	X	X	X	X	X
Adverse event reporting		X	X	X	X	X	X	X	X
Biobanking of blood/urine/tissue		X	X	X	X	X	X	X	X

### Sample size and recruitment

The aim of this study is to assess the feasibility of a large definitive trial comparing partial nephrectomy and percutaneous cryoablation in the treatment of SRM and to inform the design, sample size and outcome measures for a subsequent large multicentre prospective trial, so formal sample size calculations are inappropriate. We will aim to recruit 50 patients into the embedded randomised study, randomised equally between the two groups. This sample size will allow the assessment of whether the consent rate of 30% has been achieved with a 95% CI of ±11%. Sample sizes between 24 and 50 have been recommended to estimate the SD required for sample size calculation to allow design of a full trial.^20 21^ We anticipate being able to recruit at least 30% of eligible patients which equates to 25 patients annually, and a total recruitment time of 24 months.

Recruitment issues will be pre-empted and identified early by the qualitative researcher and their team, ensuring successful delivery of both the initial feasibility trial and full trial. This will include participant involvement in semistructured interviews and focus groups to explore views on the presentation of the study information, understanding of the trial process and reasons underlying decisions to accept or decline the trial. As part of the qualitative research effort, participants who volunteer to take part in structured interviews and focus groups will be asked to participate in additional visits. Trial management group members and clinician involvement will also take part in semistructured interviews to explore their perspectives on the study design, and their experiences with recruitment.

If interim analysis after 12 months of initiation of recruitment shows evidence of any of the following:

- consent rate of <30%,
- cryoablation results in more than a 50% increase in complications compared with partial nephrectomy at 90 days,
- the intervention results in more than a 50% increase in waiting time for treatment compared with the standard treatment group, then a detailed ‘plan of action’ will be formulated together with the qualitative research team to identify any areas in the recruitment/study pathway that can be improved. A detailed report will be presented to the Sponsor, Trial Steering Committee and the Data Monitoring Committee. If no remedial actions are deemed suitable, then the trial will be stopped.

### Trial assessments, data collection and data items

Case report forms (CRFs) will have either paper or electronic format. The CRFs will not bear the participant’s name or other directly identifiable data. The participant’s trial identification number (ID) only will be used for identification. All study-related procedures will be carried out during routine clinical visits. To maximise completeness of data, participants will also be asked for permission for research personnel to contact them via telephone. Quality control will be applied at each stage of data handling to ensure that all data are reliable and have been processed correctly. Completed CRFs will be checked for completeness and accuracy by designated individuals, against the source data. When the original CRFs are in paper format, they will be used when entering information into the computer database. The database will be checked against the CRFs for accuracy. No investigation of the data will begin until an accurate database has been assured. The CRFs will not bear the participant’s name or other directly identifiable data. The participant’s trial identification number (ID) only will be used for identification.

If a participant chooses to discontinue study participation, they should continue the follow-up schedule defined in the protocol, provided they are willing. However, if the participant confirms they do not wish to participate in the scheduled follow-up data collection visits, then data that have already been collected should be kept and analysed according to the intention-to-treat principle for all participants who stop follow-up early. Participants who stop the trial follow-up early will not be replaced.

Baseline data items include the following:

- medical and surgical history, including previous diagnosis of RCC-related hereditary syndrome, comorbidities and calculation of age corrected Charlson comorbidity index and performance status,
- current medication,
- allergies,
- physical examination, focused on abdomen (as per clinician discretion),
- baseline blood results (full blood count, renal function),
- renal tumour characteristics (complexity scoring, location, unifocality),
- body mass index, height and weight,
- occupation,
- ethnicity,
- family history of RCC or diagnosis of RCC-related hereditary syndrome,
- referral centre and date of referral,
- quality of life questionnaire (EQ-5D-5L).

For patients who will not take part in the second stage of the study (intervention), subsequent assessments will be similar in frequency and content to post-intervention assessments (except for post-procedure assessment at 4 weeks).

The following data items will be assessed at time of intervention and immediately post-procedure:

- type of anaesthesia (general, sedation and local),
- operative complications,
- adverse events (AEs) during and post-procedure,
- routine blood tests post-procedure (as per clinician discretion),
- pain score post-procedure (daily),
- length of hospital stay.

The first post-procedure assessment happens face-to-face 4 weeks after surgery. The frequency of subsequent clinical assessments will be decided based on routine clinical follow-up. Subsequent assessments can be conducted face-to-face or via telephone, as per patient and clinicians’ discretion. In the majority of patients, this will happen every 6–12 months for 5 years. In the first post-procedure and each subsequent assessment, the following will usually be assessed:

- routine blood tests (as per clinician discretion),
- physical examination, focused on abdomen (as per clinician discretion),
- cross-sectional or ultrasound imaging (as per clinician discretion),
- complications,
- pain score,
- quality of life questionnaire (EQ-5D-5L).

In addition to standard of care diagnostic samples, biological samples obtained for research purposes will be obtained at each study visit in accordance with the consent form and patient information sheet. All participants will be asked to consent for the samples to be used for current and future ethically approved research. These will include blood, urine and tissue samples. Samples will be processed and stored indefinitely in pseudo-anonymised form centrally in accordance with all applicable legal and regulatory requirements, including the Human Tissue Act 2004 and any amendments thereafter.

After the end of the trial, participants will continue follow-up as per usual care.

### Statistical methods

This is a feasibility study; therefore, all analyses should be considered exploratory. Data will be analysed on an intention-to-treat basis.

### Adverse events

All AEs will be recorded in the hospital notes in the first instance. A record of all AEs, whether related or unrelated to the treatment will also be kept in the CRF and the AE Log. The AE Log will be sent to the Sponsor on request and every 2 months. If the Investigator suspects that the disease or condition has progressed faster due to the trial intervention, this will be reported to the Sponsor. Clinically significant abnormalities in the results of objective tests (eg, laboratory variables, CT) may also be recorded as AEs.

All serious AEs will be reported both to the Sponsor within 48 hours of the investigator becoming aware of the event and via email to the relevant REC. The Chief or Principal Investigator will respond to any serious AE queries raised by the Sponsor as soon as possible. Events will be followed up until resolution; any appropriate follow-up information will be clearly marked as such and reported to the Sponsor in a timely manner. Full reports should be completed and submitted to REC within 15 days of the event.

All deaths will be reported to the Sponsor irrespective of whether the death is related to disease progression, the intervention or an unrelated event.

### Data auditing

The investigators and site will permit trial-related monitoring, audits, REC review and regulatory inspection(s), providing direct access to source data and documents. Trial participants are informed of this during the informed consent discussion. Participants will consent to provide access to their medical notes.

### Patient and public involvement

An online survey was done in collaboration with the charity Kidney Cancer UK to ascertain broad acceptability of the study’s design and willingness of patients to be recruited into NEST (results described in ‘Introduction’). This online survey suggests strong support and need from the kidney cancer patient community for the broad concepts of the study.

Two patient representatives helped draft the study protocol, patient information sheets and consent forms. One patient representative is a co-author of the present paper, will represent patient views on the trial management committee and will have a central role in the design of the full trial and dissemination of study findings.

### Ethics and dissemination

Ethical approval has been granted (UK HRA REC 19/EM/0004). Protocol amendments will be promptly disseminated via post or email to the Sponsor, all research team, Trial Management Group members, Trial Steering Committee members and Data Monitoring Committees. They will also be recorded on the trial registration website (ISRCTN18156881, registered 04/03/2019).

The Chief Investigator is the data guarantor and holder. Study outputs will be presented at national and international conferences and published in peer-reviewed journals. Professional writers will not be involved in writing the main trial report. Patient representatives will be involved in output dissemination.

10.1136/bmjopen-2019-030965.supp2 Supplementary data

10.1136/bmjopen-2019-030965.supp3 Supplementary data

10.1136/bmjopen-2019-030965.supp4 Supplementary data

10.1136/bmjopen-2019-030965.supp5 Supplementary data

## Supplementary Material

Reviewer comments

## References

[1] CRUK. Kidney cancer incidence statistics. 2015 https://www.cancerresearchuk.org/health-professional/cancer-statistics/statistics-by-cancer-type/kidney-cancer/incidence (Accessed 27 Mar 2019).

[2] Smittenaar CR, Petersen KA, Stewart K, et al Cancer incidence and mortality projections in the UK until 2035. Br J Cancer 2016;115:1147–55. 10.1038/bjc.2016.304 27727232 PMC5117795

[3] Homma Y, Kawabe K, Kitamura T, et al Increased incidental detection and reduced mortality in renal cancer--recent retrospective analysis at eight institutions. Int J Urol 1995;2:77–80. 10.1111/j.1442-2042.1995.tb00428.x 7553292

[4] Luciani LG, Cestari R, Tallarigo C Incidental renal cell carcinoma-age and stage characterization and clinical implications: study of 1092 patients (1982-1997). Urology 2000;56:58–62. 10.1016/S0090-4295(00)00534-3 10869624

[5] Nayyar M, Cheng P, Desai B, et al Active surveillance of small renal masses: a review on the role of imaging with a focus on growth rate. J Comput Assist Tomogr 2016;40:517–23. 10.1097/RCT.0000000000000407 27331922

[6] Jewett MA, Mattar K, Basiuk J, et al Active surveillance of small renal masses: progression patterns of early stage kidney cancer. Eur Urol 2011;60:39–44. 10.1016/j.eururo.2011.03.030 21477920

[7] Ljungberg B, Albiges L, Abu-Ghanem Y, et al European association of urology guidelines on renal cell carcinoma: the 2019 update. Eur Urol 2019;75:799–810. 10.1016/j.eururo.2019.02.011 30803729

[8] Fernando A, Fowler S, O’Brien T Nephron-sparing surgery across a nation - outcomes from the British Association of Urological Surgeons 2012 national partial nephrectomy audit. BJU Int 2016;117:874–82. 10.1111/bju.13353 26469291

[9] NHS England. Robot-assisted nephrectomy. 2014 https://www.engage.england.nhs.uk/consultation/clinical-commissioning-wave8/user_uploads/b14x12-evidence-rev.pdf (Accessed 28 Mar 2019).

[10] Xing M, Kokabi N, Zhang D, et al Comparative effectiveness of thermal ablation, surgical resection, and active surveillance for T1a renal cell carcinoma: a surveillance, epidemiology, and end results (SEER)-Medicare-linked Population Study. Radiology 2018;288:81–90. 10.1148/radiol.2018171407 29737950

[11] Thompson RH, Atwell T, Schmit G, et al Comparison of partial nephrectomy and percutaneous ablation for cT1 renal masses. Eur Urol 2015;67:252–9. 10.1016/j.eururo.2014.07.021 25108580

[12] Klatte T, Shariat SF, Remzi M Systematic review and meta-analysis of perioperative and oncologic outcomes of laparoscopic cryoablation versus laparoscopic partial nephrectomy for the treatment of small renal tumors. J Urol 2014;191:1209–17. 10.1016/j.juro.2013.11.006 24231845

[13] Astani SA, Brown ML, Steusloff K Comparison of procedure costs of various percutaneous tumor ablation modalities. Radiol Manage 2014;36:12–17. quiz 18-9. 25174139

[14] NCRI. NCRI bladder & renal cancer clinical studies group. 2018 http://csg.ncri.org.uk/wp-content/uploads/2018/10/NCRI-Bladder-Renal-Cancer-CSG-2017-2018-Annual-Report.pdf (Accessed 28 Mar 2019).

[15] Beekman E, Mesters I, Hendriks EJ, et al Exacerbations in patients with chronic obstructive pulmonary disease receiving physical therapy: a cohort-nested randomised controlled trial. BMC Pulm Med 2014;14:71 10.1186/1471-2466-14-71 24767519 PMC4108017

[16] Burbach JP, Kurk SA, Coebergh van den Braak RR, et al Prospective Dutch colorectal cancer cohort: an infrastructure for long-term observational, prognostic, predictive and (randomized) intervention research. Acta Oncol 2016;55:1273–80. 10.1080/0284186X.2016.1189094 27560599

[17] Kim SY, Flory J, Relton C Ethics and practice of trials within cohorts: an emerging pragmatic trial design. Clin Trials 2018;15:9–16. 10.1177/1740774517746620 29224380 PMC6006508

[18] Donovan J, Mills N, Smith M, et al Quality improvement report: Improving design and conduct of randomised trials by embedding them in qualitative research: ProtecT (prostate testing for cancer and treatment) study. Commentary: presenting unbiased information to patients can be difficult. BMJ 2002;325:766–70. 10.1136/bmj.325.7367.766 12364308 PMC1124277

[19] Chan AW, Tetzlaff JM, Gøtzsche PC, et al SPIRIT 2013 explanation and elaboration: guidance for protocols of clinical trials. BMJ 2013;346:e7586 10.1136/bmj.e7586 23303884 PMC3541470

[20] Julious SA Sample size of 12 per group rule of thumb for a pilot study. Pharm Stat 2005;4:287–91. 10.1002/pst.185

[21] Sim J, Lewis M The size of a pilot study for a clinical trial should be calculated in relation to considerations of precision and efficiency. J Clin Epidemiol 2012;65:301–8. 10.1016/j.jclinepi.2011.07.011 22169081

