# Supplementary material for: Protocol for a feasibility study of a cohort embedded randomised controlled trial comparing NEphron Sparing Treatment (NEST) for small renal masses
Source: BMJ Open. 2019 Jun 11;9(6):e030965. doi: 10.1136/bmjopen-2019-030965 (PMC6577353; doi:10.1136/bmjopen-2019-030965)
Supplement: Supplementary data [file bmjopen-2019-030965supp002.pdf]

PATIENT CONSENT FORM

NEST Cohort Study

A feasibility study of a cohort embedded randomised control trial comparing NEphron Sparing Treatment for small renal masses (NEST)

Chief Investigator: **Miss Maxine Tran**

	Please initial the box to agree	
1. I confirm that I have read the information sheet dated .... (version ....) for the above study. I have had the opportunity to consider the information, ask questions and have had these answered satisfactorily.		
2. I understand that my participation is voluntary and that I am free to withdraw at any time without giving any reason, without my medical care or legal rights being affected.		
3. I understand that relevant sections of my medical notes and data collected during the study may be looked at by individuals from the Sponsor of this study, from regulatory authorities or from the NHS Trust, where it is relevant to my taking part in this research. I give permission for these individuals to have access to my records.		
4. I understand that the information collected, including imaging scans (for example, ultrasounds, CTs and MRI), about me will be used to support other research in the future, and may be shared anonymously with other researchers, including collaborative research groups which may not be based at the Royal Free London NHS Foundation Trust.		
5. I understand that the anonymised results of this research could be stored indefinitely in an electronic archive. I understand that this archive will be accessible to researchers worldwide who will use the results to advance scientific and medical understanding.		
6. I understand that the information held and maintained by Royal Free London NHS Foundation Trust and NHS Digital may be used to help contact me or provide information about my health status.		
7. I agree to be invited to participate in studies testing new treatments for small renal masses if I am suitable. *(Optional)*	YES	NO
8. I agree to my data and results being used for comparative purposes in this research study.		

9. I agree to my blood and urine being collected, stored and analysed for this research study, which may include DNA and the growing/establishing of live cells.	
10. I agree to tissue samples obtained from biopsy being collected, stored and analysed for this research study, which may include DNA and the growing/establishing of live cells.	
11. I agree to tissue samples obtained from surgery being collected, stored and analysed for this research study, which may include DNA and the growing/establishing of live cells.	
12. I agree to my blood, urine and tissue samples being analysed for future ethical approved research studies, which may include DNA and the growing/establishing of live cells. *(Optional)*	YES / NO
13. I agree to complete the questionnaires about my quality of life after diagnosis and treatment of small renal mass.	
14. I understand that I may not benefit directly or financially from this study.	
15. I agree to my General Practitioner being informed of my participation in the Study, including any necessary exchange of information about me between my GP and the research team.	
16. I agree to part take in this Research Project.	

______________________	___________	______________________
Name of patient	Date	Signature
______________________	___________	______________________
Name of Person taking consent	Date	Signature
______________________	___________	______________________
Co-sign (if applicable)	Date	Signature

To be filled out by research team:

Centre Number:

Study Number:

Participant Identification Number for this trial:
