# Supplementary material for: Protocol for a feasibility study of a cohort embedded randomised controlled trial comparing NEphron Sparing Treatment (NEST) for small renal masses
Source: BMJ Open. 2019 Jun 11;9(6):e030965. doi: 10.1136/bmjopen-2019-030965 (PMC6577353; doi:10.1136/bmjopen-2019-030965)
Supplement: Supplementary data [file bmjopen-2019-030965supp003.pdf]

PATIENT CONSENT FORM

NEST Interventional Study

A feasibility study of a cohort embedded randomised control trial comparing NEphron Sparing Treatment for small renal masses (NEST)

Chief Investigator: Miss Maxine Tran

	Please initial the box to agree
1. I confirm that I have read the information sheet dated .... (version ....) for the above study. I have had the opportunity to consider the information, ask questions and have had these answered satisfactorily.	
2. I understand that my participation is voluntary and that I am free to withdraw at any time without giving any reason, without my medical care or legal rights being affected.	
3. I understand that relevant sections of my medical notes and data collected during the study may be looked at by individuals from the Sponsor of this study, from regulatory authorities or from the NHS Trust, where it is relevant to my taking part in this research. I give permission for these individuals to have access to my records.	
4. I understand that the information collected about me, including imaging scans (for example, ultrasounds, CTs and MRI), will be used to support other research in the future and may be shared anonymously with other researchers, including collaborative research groups which may not be based at the Royal Free London NHS Foundation Trust.	
5. I agree to my General Practitioner being informed of my participation in the Study, including any necessary exchange of information about me between my GP and the research team.	
6. I understand that the information held and maintained by Royal Free London NHS Foundation Trust and NHS Digital may be used to help contact me or provide information about my health status.	
7. I agree to have cryotherapy treatment for my small renal mass.	
8. I agree to part take in this Research Project.	

___________________________	______________	__________________________
Name of patient	Date	Signature

___________________________	______________	__________________________
Name of Person taking consent	Date	Signature

Co-sign (if applicable)	Date	Signature

To be filled out by research team:

Centre Number:

Study Number:

Participant Identification Number for this trial:
