# Supplementary material for: Protocol for a feasibility study of a cohort embedded randomised controlled trial comparing NEphron Sparing Treatment (NEST) for small renal masses
Source: BMJ Open. 2019 Jun 11;9(6):e030965. doi: 10.1136/bmjopen-2019-030965 (PMC6577353; doi:10.1136/bmjopen-2019-030965)
Supplement: Supplementary data [file bmjopen-2019-030965supp004.pdf]

PATIENT INFORMATION SHEET

NEST Cohort Study

A feasibility study of a cohort embedded randomised control trial comparing NEphron Sparing Treatment for small renal masses (NEST)

Chief Investigator: **Miss Maxine Tran**

We would like to invite you to take part in the above research study. Before you decide whether you want to take part, it is important that you understand why the study is being done and what participating in the study entails. One of our team will go through this information sheet with you, to help you decide whether you would like to take part or not and answer any questions you may have. We'd suggest this should take about 45 minutes. Please read the following information carefully and please feel free to talk to others about the study if you wish.

What is the purpose of the study?

This is a clinical research study that invites adult men and women who have been diagnosed with a small renal mass (that is, a kidney lump that measures 4cm or less) to be followed up long term (at least 5 years after diagnosis).

The primary purpose of the study is to collect and analyse clinical information, as well as blood, urine, and tissue samples, that will allow us to find characteristics that determine why some renal tumours grow, why some tumours don't grow, and why some get smaller. The other purpose of the study is to establish a group (cohort) of which some patients could be suitable for new treatment interventions. These patients can be selected and may be offered the new treatment if suitable, instead of the current standard of care treatment.

Why is the study being done?

Kidney cancer is diagnosed in over 14000 patients every year in the UK and is one of the few cancers where the number of cases diagnosed each year is predicted to increase for the next 20 years. At least 1 in 3 kidney cancers are diagnosed at a stage when they measure 4cm or less. After diagnosis, some of these small cancers will grow, some will not grow, and some may even shrink without treatment. Currently, we are not able to predict individual tumour behaviour. By following up a large population of patients for a long period of time we hope to identify characteristics that will enable us to differentiate which groups of patients require aggressive intervention from patients for whom it is safe to just monitor tumours over time and therefore avoid unnecessary treatment. Additionally, we also want to study if new treatment options can be offered to patients diagnosed with a small renal mass.

Why have we invited you to take part in this study?

We have invited you to take part in this study because you have been diagnosed with a small renal mass and referred to the Specialist Centre for Kidney Cancer at Royal Free London NHS Foundation Trust.

Do I have to take part?

No, participating in this study is voluntary and you only take part if you wish. If you prefer not to take part in the study, this will not affect the current care you receive from doctors, nurses, or any other healthcare professionals.
If you agree to take part in the study, you can change your mind about participation in the study at any time, and this will not affect your current or future medical care.

What will happen to me if I take part in the study?

If you decide to participate in the study, you will be asked to sign the study consent form and we will ask you to:

Donate blood and urine samples:

- When you first come to clinic after agreeing to take part in the study
- When you have treatment (for example, surgery)
- When you have follow-up appointments (usually at the same time as your clinic consultation to minimise inconvenience to yourself) for at least 5 years after diagnosis. The first post-procedure follow up happens face-to-face 4 weeks after surgery. Future assessments will be decided based on routine clinical follow up. Subsequent assessments can be conducted face-to-face or via telephone, as per patient and clinicians' discretion. For the majority of patients this will happen 6 to 12 monthly, for 5 years.

Donating these samples will usually add 10 to 15 minutes to each clinic appointment. We will ask you to donate no more than 50 ml of blood, which is approximately 4 tablespoons and up to 30 ml of urine, which is about 2 tablespoons.

Donate tissue samples:

If you are having a biopsy of your tumour, the doctor taking the biopsy may take additional biopsies of the kidney and tumour for this research. This may not be possible if the tumour is small or if there is little tissue for diagnostic purposes. Taking additional biopsies carry a (presumed) small increased risk for bleeding which is not quantifiable, but this will not change the usual post-biopsy care and advice.

If you are having surgery, once the tumour or kidney is removed, a doctor will take samples for your diagnosis. Some of the tissue that is left over will be stored for research purposes. Because taking these samples will not affect how surgery is done, there are no additional operative risks for you.

Fill out questionnaires:

This will allow us to understand how being diagnosed, treated, and/or followed up for a small renal mass has impacted you, your day to day activities, and your quality of life. These questionnaires will be given to you when you come to clinic, sent to you via post or via electronic mail. We will ask you to fill out questionnaires for at least 5 years after diagnosis. Filling out each questionnaire will usually take between 15 minutes and 1 hour.

We will also collect data relating to your clinical management, such as past medical history, details of how and when you were diagnosed with a kidney lump, laboratory results, or images from any scans (ultrasound, CT, MRI). Some of the analyses of this data may be performed by other research teams that we work with, if we transfer your images out of the Royal Free London NHS Foundation Trust, it will be in an anonymised form, so you will not be identifiable.

We will ask for your consent to compare your data, that has been collected during this study, with other participants in the cohort and randomised control trial.

We will also ask for your consent to link your data to NHS Digital records or other NHS registries to find out how your health is in the future and to be able to contact you, should you be 'lost to follow-up'.

Finally, we will ask you if you would be interested in participating in studies that test new or alternative treatments or tests for kidney cancer. If you are interested, we will include you in the pool of patients that are suitable for new or alternative treatments or tests. Patients from this pool will be randomly selected to receive invitations to consider the new or alternative treatment or test, if suitable. If we invite you, you will receive written information and come to clinic to learn more about it. We will only ask you to consent to having the new or alternative treatment/test after giving you all the information you require to make an informed decision. If we don't invite you for the new or alternative treatment/test, you would continue with standard routine treatment/tests as advised by your doctor.

What are the possible risks and unwanted side effects of the study?

There are no significant risks associated with giving a urine sample or with giving tissue that has already been removed by means of surgery. There may be some bruising associated with providing a blood sample, and very occasionally, patients may feel faint, dizziness, or discomfort.
If you and your doctor have decided that you should have surgery, this can be done either by removing just the tumour (partial nephrectomy) or by removing the whole kidney (radical nephrectomy). Surgery itself is associated with risks but because tissue samples will be taken after the surgery is completed, taking these samples not affect how surgery is done and there are no additional operative risks for you. Some of the risks of surgery include bleeding, injury to surrounding structures (for example, bowel, blood vessels, liver, spleen), pain or discomfort after surgery, anaesthetic risk, urine infection, renal failure, among others. The actual risks and how many patients are on average affected by these risks depend on a number of variables, including type of surgery, how it will be done (robotic, laparoscopic or via open approach), and, for example, your kidney function before surgery and where the tumour is located. Please discuss with your doctor the risks of surgery that are applicable to your situation.

The risks associated with having a biopsy include pain, discomfort, soreness, redness and infection. Serious complications such as bleeding requiring blood transfusion or other intervention can occur in 0.5% of patients (1 in 200). The biopsy will be done using ultrasound or CT guidance, according to what has already been decided by the clinical team. Taking additional biopsies carry a (presumed) small increased risk for bleeding which is not quantifiable, but this will not change the usual post-biopsy care and advice.

For this study, we will not ask you to have additional imaging scans. However, you may have CT scans done as part of your usual clinical care following a diagnosis of a small renal mass. These scans, as well as having a biopsy under CT guidance, include exposure to radiation.

If you are a woman and may be pregnant, radiation exposure can be associated with risk of harm to an unborn child. It can also carry risk when breast-feeding. Any women having a CT scan should have a pregnancy test done prior to the scan. There are no contraceptive requirements to take part in the study. If you become pregnant during the study, you should inform the study team using the contacts stated below, as well as the clinical team if any CT scans have been scheduled as part of your usual clinical care. Should you become pregnant, the clinical team will decide if it is more appropriate for you to have other types of scans that are not associated with radiation, such as ultrasound scans. Your pregnancy care will be monitored as clinically required, no additional monitoring will be done by the research team.

If you are a man, radiation exposure can be associated with sperm damage and consequently pose a risk to possible pregnancies. If you are concerned about this and are having CT scans as part of your usual clinical care, please discuss with your doctor if other scans that are not associated with radiation can be undertaken, such as ultrasound scans.

What will happen with the samples taken for research purposes?

Samples will be used to study new blood, urine and/or tissue markers of small renal masses that will enable improved and more accurate diagnosis. Analysis of the samples may include testing of the cells, of molecules produced by the cells, and/or genetic material (such as DNA).

All samples donated will be stored in the UCL/Royal Free BioBank and/or in the laboratories of the research projects that have been granted ethical approval to use the samples. Only ethically approved researched projects will also be able to use these samples. This information will not identify you and will not be combined with other information in a way that could identify you. The information will only be used for the purpose of health and care research and cannot be used to contact you or to affect your care. It will not be used to make decisions about future services available to you, such as insurance.

What about patient confidentiality?

Samples will be given a unique code so that laboratory researchers receiving the samples do not know your name or personal details. Samples can only be traced back to the patient by the clinical team; samples will otherwise be kept anonymous. Tissue may be transferred to other external organisations under written agreement, which guarantees the use and safe keeping of the samples.
All the data we collect from you will be given a unique code so that researchers do not know your name or personal details. The code can only be traced back to the patient by the clinical team or your GP; data will otherwise be kept anonymous.

Royal Free London NHS Foundation Trust is the sponsor for this study based in the United Kingdom. We will be using information from you and or your medical records in order to undertake this study and will act as the data controller for this study. This means that we are responsible for looking after your information and using it properly. The Trust will keep identifiable information about you for 5 years after the study has finished.
Your rights to access, change or move your information are limited, as we need to manage your information in specific ways in order for the research to be reliable and accurate. If you withdraw from the study, we will keep the information about you that we have already obtained. To safeguard your rights, we will use the minimum personally-identifiable information possible.

You can find out more about how we use your information at: https://www.royalfree.nhs.uk/patients-visitors/how-we-use-patient-information

The Trust, as sponsor, will act as data controllers for this study. University College London (UCL) will act as the data processors on behalf of the sponsor for collected data for the research. The data will be stored in UCL Safe Haven.

We will keep your name, NHS number and contact details confidential and will not pass this information on. We will use this information as needed, to contact you about the research study, and make sure that relevant information about the study is recorded for your care, and to oversee the quality of the study. Certain individuals from Royal Free London NHS Foundation Trust and regulatory organisations may look at your medical and research records to check the accuracy of the research study. The people who analyse the information will not be able to identify you and will not be able to find out your name, NHS number or contact details.

Do I need to agree to everything in the study?

No. If you feel uncomfortable with answering certain questions or do not want to have certain measures or to give a blood sample, you do not have to do so. You can still participate in the other parts of the study.

What are the possible benefits to me and for others of me taking part?

This study is unlikely to be of immediate personal or financial benefit to you. We hope that this study will enable us to develop better diagnostic, prognostic, and treatment strategies for the future management of kidney cancer.

Involvement of your General Practitioner (GP)/family doctor

With your permission, your GP will be informed of your participation in this study. However, your normal care pathway will not be affected and if for any reason you would not like your GP to know, this will be respected.

What if I change my mind?

You can change your mind at any time and withdraw consent for the study. This will not affect your current or future clinical care.

If clinical information, tissue, blood or urine samples have already been used in research, then the anonymised results may be stored indefinitely in an electronic archive. This archive will be accessible to *bona fide* researchers worldwide who will use the results to advance scientific and medical understanding. Anonymised data cannot be retrieved to be deleted. If your clinical information, tissue, blood or urine samples have not yet been used, then the samples will be destroyed/incinerated in the usual protocol for disposal of human materials

What will happen to the results of the research study?

When the study is completed the results will be analysed and presented at national and international meetings before being published in a medical journal. The results of this study will not influence your usual clinical care. If you wish to receive information on these results when they are presented, please ask your study doctor.

What if something goes wrong?

In the event that something does go wrong, and you are harmed during the research and this is due to someone's negligence then you may have grounds for a legal action for compensation against Royal Free London NHS Foundation Trust, but you may have to pay your legal costs. The normal National Health Service complaints mechanisms will still be available to you.

Complaints

If you have a concern about any aspect of this study, you should ask to speak to the researchers who will do their best to answer your questions. If you remain unhappy and wish to complain formally, you can do this by contacting the confidential patient advice and liaison service (PALS). PALS was set up to support patients, their families and visitors who need advice or have problems and concerns.

The patient advice and liaison service for the Royal Free Hospital is in the hospital’s main reception. The service is open from 10am to 4pm, Monday to Friday, except Wednesday, when the service is open from 10.30am to 4pm.
Tel: 020 7472 6446/6447; (020 7472 6445 - 24-hour answer phone)
Fax: 020 7472 6463
SMS: +447860023323 (Deaf and hearing-impaired patients only)
Email: rf.pals@nhs.net

Who is organising and funding the research?

Royal Free London NHS Foundation Trust is the sponsor for the study. The National Institute of Health Research (NIHR) and Facing up 2 Kidney cancer is funding the research. The study is being conducted at the Specialist Centre for Kidney Cancer at the Royal Free London NHS Foundation Trust. Miss Maxine Tran is the Chief Investigator of the study.

How have patients and the public been involved in this study?

Ninety-nine patients and members of the public responded to an online questionnaire hosted by Kidney Cancer UK over a two-week period. Almost all of the responses were in favour and supportive of the general aims and concepts of the study. There are also two patient representatives on the study committee, who have helped in the drafting of this information sheet, consent form and study protocol. The patient representatives will also be attending the regular trial meetings to monitor the progress of the study. They will also be helping with disseminating the results of the study at patient education days, national conferences and online platforms.

Who has reviewed the study?

All research in the NHS is looked at by an independent group of people, called a Research Ethics Committee, to protect your interests. This study has been reviewed and given favourable opinion by _________Research Ethics Committee. This study has also been reviewed by the Research for Patient Benefit Programme at the National Institute of Health Research (NIHR), which has agreed to fund the study.

Contacts for further information

If you would like further information or have any questions about this study please consult your consultant or contact us on the number below. You may also find it useful to contact cancer BACUP, an independent patient advisory group (Freephone 0800181199).

Mr David Cullen
Lead Nurse Renal cancer
07775687823

Miss Sara Hamilton
Lead Cancer Research Nurse
0207 794 0500
Sara.hamilton@nhs.net

Thank you for taking the time to read the information about the study.
