# Supplementary material for: Protocol for a feasibility study of a cohort embedded randomised controlled trial comparing NEphron Sparing Treatment (NEST) for small renal masses
Source: BMJ Open. 2019 Jun 11;9(6):e030965. doi: 10.1136/bmjopen-2019-030965 (PMC6577353; doi:10.1136/bmjopen-2019-030965)
Supplement: Supplementary data [file bmjopen-2019-030965supp005.pdf]

PATIENT INFORMATION SHEET

Invitation to: Treatment of Small Renal Mass Using Percutaneous Cryoablation

A feasibility study of a cohort embedded randomised control trial comparing NEphron Sparing Treatment for small renal masses (NEST)

Chief Investigator: **Miss Maxine Tran**

We would like to invite you to take part in the above research study. Before you decide whether you want to take part, it is important that you understand why the study is being done and what participating in the study entails. One of our team will go through this information sheet with you, to help you decide whether you would like to take part or not and answer any questions you may have. We'd suggest this should take about 45 minutes. Please read the following information carefully and please feel free to talk to others about the study if you wish.

What is the purpose of the study?

This is a clinical research study that invites adult men and women who have been diagnosed with a small renal mass (that is, a kidney lump that measures 4cm or less) to have it treated using percutaneous cryoablation (killing of the tumour cells by means of freezing using special needles that are inserted through the skin). This is a NICE approved and NHS funded treatment option for kidney cancer.

The primary purpose of the study is to understand if patients would be willing to be offered the option of having a new treatment for their small renal mass, called cryoablation, instead of the standard of care option, partial nephrectomy.

Why is the study being done?

This study is being done for two reasons. In the past, it has been difficult to recruit patients into traditional clinical trials that compare two or more surgical treatment/s. This has happened for a number of reasons, including unwillingness of patients to have a treatment that has been randomly chosen for them and tendency of doctors to encourage patients to choose to have one treatment instead of the other. The design of the study we are running (recruiting patients first to be followed up over a period of time and, from then on, randomly inviting them to be offered to have a new or alternative intervention) has been shown to be very effective at recruiting patients and studying new treatment options for other diseases. As a first step, we want to prove that it can be effective to also study new or alternative treatment options in kidney cancer.

Currently the standard of care treatment for a small renal mass is a type of surgery called partial nephrectomy. This is surgery to remove the kidney lump while leaving the remaining kidney in place. This has been shown to be as effective for cancer control as taking out the whole kidney, with the likely advantage of reducing the likelihood of having renal failure in the future (by preserving more kidney tissue instead of leaving patients with just one kidney). However, partial nephrectomy is a complex and demanding surgery, only suitable for certain patients who are fit from the anaesthetic point of view. In patients unfit for partial nephrectomy, a different treatment modality has been used for at least 10 years called cryoablation. Cryoablation involves freezing off just the kidney tumour, leaving the remaining kidney intact. Studies suggest that using cryoablation in this population of patients provides good cancer control with additional advantages over partial nephrectomy, including faster recovery after surgery, less operative complications, and more preservation of kidney function. For these reasons, we want to conduct a study to compare the two treatments (partial nephrectomy and cryoablation). Before we do so, we want to find out if patients would be willing to participate in a study that aims to do such a comparison.

Why have we invited you to take part in this study?

You have been invited to take part in this study for several reasons:

- You have been diagnosed with a small renal mass and referred to the Specialist Centre for Kidney Cancer at Royal Free London NHS Foundation Trust.
- You have previously agreed to participate in a study that follows up patients with small renal masses over time and to allows participants to be randomly invited to be offered new or alternative treatment options if they are suitable.
- You have a had a tumour biopsy that has confirmed you have kidney cancer;
- You are equally eligible to have the standard of care treatment (partial nephrectomy) and the alternative intervention, called cryoablation.
- You have been randomly selected to be invited to consider having your small renal mass treated using cryoablation.

Do I have to take part?

No, participating in this study is voluntary and you only have to take part if you wish. If you prefer not to take part in the study, this will not influence affect the clinical current care you receive from doctors, nurses, or any other healthcare professionals.

If you agree to take part in the study, you can change your mind about participation in the study at any time, and this will not affect your current or future medical care.

If you choose not to have cryoablation of your kidney tumour, you will be offered the standard of care treatment, partial nephrectomy.

Whether you decide to participate in the study or not, we may contact you to be part of semi-structured interviews (to be done in person or over the telephone) or part of a focus group to explore your views and opinions about the trial procedures. This will be help us understand how we can improve and optimise the way the trial is being done. Each semi-structured interview should take roughly 30-60 mins and you can take part in up to 3 interviews. The focus group should last for a maximum of 2h, with breaks. Again, this is voluntary and you only have to take part if you wish. The interviews and focus groups will be audio recorded, and the files will be saved on secure UCL computers which will be password protected. The interviews and focus group comments will then be transcribed and analysed by the research team. Audio recordings will be destroyed once transcription and analysis has been completed.

What will happen to me if I take part in the study?

If you decide to participate in the study, you will be asked to sign the study consent form and you will have cryoablation to treat your kidney tumour. The cryoablation procedure will be conducted at University College London Hospital. Before treatment, you will have to have an appointment with the anaesthetic team that will include blood and urine tests. Other studies may be required pre-operatively to decide if you are fit to be given general anaesthetic, such as an echocardiogram. These will be decided on a patient by patient basis. We will also ask you to fill out some questionnaires before you have treatment. Filling out each questionnaire will usually take between 15 min and 1 hour.

On the day of surgery, your tumour will be treated by freezing the lesion. This procedure is usually carried out by a interventional radiologist. This happens in a special theatre room equipped with a CT scanner. Images of your tumour will be taken to allow for the correct placement of the freezing needles and to monitor treatment. A number of needles (usually ranging from 3 to 7) will be inserted from the back, through the skin into the tumour. The tip of each needle

is brought to a very low temperature level that allows for an ice ball to be created. The ice balls created by all the needles is large enough to cover the whole lesion. After the procedure, the needles are removed. Because the procedure is done under CT guidance, this involves exposure to radiation.

After treatment, you will be followed up regularly in clinic. The first visit in clinic will happen at 4 to 6 weeks after your treatment. Because you have previously consented to be followed up for long term, we will continue to ask you to fill out questionnaires after treatment, as well as donate blood and urine samples for research when you come to clinic.

What are the possible disadvantages and unwanted side effects of the study?

In the following tables, we explain the main differences and possible side effects of partial nephrectomy (the standard of care treatment) and percutaneous cryoablation (the treatment option you have been invited to have).

	**Partial nephrectomy**	**Percutaneous cryoablation**
**Method**	Robot assisted keyhole surgery to remove the kidney tumour	Freezing of tumour using needles that are inserted through the skin into the kidney tumour
**Surgical wounds**	4 to 5 small wounds around 1cm and 1 larger wound around 3 to 4cm, all closed with surgical staples and covered with small dressings/plaster	3 to 7 puncture sites (less than 0.5cm), covered with small dressing/plaster
**Anaesthetic**	General anaesthetic	General anaesthetic
**Recovery after surgery**	Home 2 days after surgery. Usually it takes 4 weeks to fully recover and be able to perform all day to day activities.	Home one day after surgery. Usually it takes 2 weeks to fully recover and be able to perform all day to day activities.
**Cancer control**	Proven to provide good cancer control in all patients.	Proven to provide good cancer control in older patients and in patients not fit for surgery.<br>This study will investigate cancer control in other patients.

Possible side effects	How often will they happen	
	Partial nephrectomy	Cryoablation
**Bleeding that requires a blood transfusion and/or a further procedure** (embolisation)	Less than 1 in 10 patients	1 in 100 patients
**Injury to surrounding structures, including bowel or blood vessels**	Less than 1 in 50 patients	Less than 1 in 100 patients
**Leak of urine at the cut edge of the kidney**	Less than 1 in 50 patients	Less than 1 in 200 patients
**Pain or discomfort after surgery**	Most patients, including temporary pain in the tip of the shoulder, and temporary bloating of the tummy	Most patients (mostly discomfort)
**Renal failure**	Possible (risk depends on baseline renal function)	Possible (risk depends on baseline renal function)
**Infection** (urine, kidney, chest, wounds)	Possible	Possible
**Under treatment of cancer** (if this happens, you may need further treatment)	1 in 30 patients	1 in 30 patients
**Recurrence of cancer**	up to 1 in 25 patients	1 in 30 patients
**Removal of the whole kidney at time of procedure**	In less than 1 in 10 patients this can happen if removing just the tumour is found technically not possible at time of surgery	Never
**Conversion to open surgery**	Less than 1 in 10 patients	Never
**Skin numbness and/or bulging**	Unlikely	Less than 1 in 10 patients (may be irreversible in 1 in 200 patients).
**Post-ablation syndrome** (flu-like illness that happens 3-5 days after treatment)	Unlikely	1 in 4 patients.<br>In less than 1 in 100 patients, re-admission or prolonged admission may be required.

**Anaesthetic or cardiovascular risks** (chest infection, clots in legs and lungs, stroke, heart attack, death)	Unlikely but possible	Unlikely but possible
**Radiation risk**	No	Yes (please see below)

For this study, we will not ask you to have any additional imaging scans. However, you may have CT scans done as part of your usual clinical care following a diagnosis of a small renal mass, and the cryoablation procedure itself involves CT scanning. The biopsy may also be carried out under CT guidance. CT scans use ionising radiation to form images of your body and provide your doctor with other clinical information. Ionising radiation can cause cell damage that may, after many years or decades, turn cancerous. The chances of this happening to you are the same whether you take part in this study or not.

If you are a woman and may be pregnant, radiation exposure can be associated with risk of harm to an unborn child. It can also carry risk when breast-feeding. Any women having a CT scan should have a pregnancy test done prior to the scan. If you are pregnant or breast-feeding at the time the cryoablation is planned to take place, this procedure should not be performed and you should be offered another management modality, such as the standard of care (partial nephrectomy) or active surveillance until safe delivery of the baby or end of the breast-feeding period. If you become pregnant during the study, you should inform the study team using the contacts stated below, as well as the clinical team. If you become pregnant or start breast-feeding after treatment has been done and have follow-up CT scans booked as part of your usual clinical care, the clinical team will decide if it is more appropriate for you to have other types of scans that are not associated with radiation, such as ultrasound scans. Your pregnancy care will be monitored as clinically required, no additional monitoring will be done by the research team.

If you are concerned about radiation dose and are having CT scans as part of your usual clinical care after the cryoablation, please discuss with your doctor as other scans that are not associated with radiation may be undertaken, such as MRI scans.

What about patient confidentiality?

All the data we collect from you will be given a unique code so that researchers do not know your name or personal details. The code can only be traced back to the patient by the clinical team or your GP; data will otherwise be kept anonymous.

Royal Free London NHS Foundation Trust is the sponsor for this study based in the United Kingdom. We will be using information from you and or your medical records in order to undertake this study and will act as the data controller for this study. This means that we are responsible for looking after your information and using it properly. The Trust will keep identifiable information about you for 5 years after the study has finished.
Your rights to access, change or move your information are limited, as we need to manage your information in specific ways in order for the research to be reliable and accurate. If you withdraw from the study, we will keep the information about you that we have already obtained. To safeguard your rights, we will use the minimum personally-identifiable information possible.

You can find out more about how we use your information at: https://www.royalfree.nhs.uk/patients-visitors/how-we-use-patient-information

The Trust, as sponsor, will act as data controllers for this study. University College London (UCL) will act as the data processors on behalf of the sponsor for collected data for the research. The data will be stored in UCL Safe Haven.

We will keep your name, NHS number and contact details confidential and will not pass this information on. We will use this information as needed, to contact you about the research study, and make sure that relevant information about the study is recorded for your care, and to oversee the quality of the study. Certain individuals from Royal Free London NHS Foundation Trust and regulatory organisations may look at your medical and research records to check the accuracy of the research study. The people who analyse the information will not be able to identify you and will not be able to find out your name, NHS number or contact details.

What are the possible benefits to me and for others of me taking part?

We hope that cryoablation will help you, that you will experience less side effects from treatment than you would if you had a partial nephrectomy, and that, because cryoablation is a less invasive treatment, your recovery after surgery will be quicker and you will be able to go back sooner to your normal day to day life. However, this cannot be guaranteed as we don't know what the outcome of the study will be. This why we are conducting this research; information gathered from this study will hopefully help us treat future patients diagnosed with a small renal masses better.

Involvement of your General Practitioner (GP)/family doctor

With your permission, your GP will be informed of your participation in this study. However, your normal care pathway will not be affected and if for any reason you would not like your GP to know, this will be respected.

What if I change my mind?

You can change your mind at any time and withdraw consent for the study. This will not affect your current or future clinical care.

If clinical information, tissue, blood or urine samples have already been used in research, then the anonymised results may be stored indefinitely in an electronic archive. This archive will be accessible to *bona fide* researchers worldwide who will use the results to advance scientific and medical understanding. Anonymised data cannot be retrieved to be deleted. If your clinical information, tissue, blood or urine samples have not yet been used, then the samples will be destroyed/incinerated in the usual protocol for disposal of human materials

What will happen to the results of the research study?

When the study is completed the results will be analysed and presented at international meetings before being published in a medical journal. The results of this study will not influence your usual clinical care. If you wish to receive information on these results when they are presented, please ask your study doctor.

What if something goes wrong?

In the event that something does go wrong, and you are harmed during the research and this is due to someone's negligence then you may have grounds for a legal action for compensation against Royal Free London NHS Foundation Trust, but you may have to pay your legal costs. The normal National Health Service complaints mechanisms will still be available to you.

Complaints

If you have a concern about any aspect of this study, you should ask to speak to the researchers who will do their best to answer your questions. If you remain unhappy and wish to complain formally, you can do this by contacting the confidential patient advice and liaison service (PALS). PALS was set up to support patients, their families and visitors who need advice or have problems and concerns.

The patient advice and liaison service for the Royal Free Hospital is in the hospital's main reception. The service is open from 10am to 4pm, Monday to Friday, except Wednesday, when the service is open from 10.30am to 4pm.
Tel: 020 7472 6446/6447; (020 7472 6445 - 24-hour answer phone)
Fax: 020 7472 6463
SMS: +447860023323 (Deaf and hearing-impaired patients only)
Email: rf.pals@nhs.net

Who is organising and funding the research?

Royal Free London NHS Foundation Trust is the sponsor for the study. Facing up 2 Kidney cancer is funding the study as well as The National Institute for Health Research is funding the research through its Research for Patient Benefit Programme. The study is being conducted at the Specialist Centre for Kidney Cancer at the Royal Free London NHS Foundation Trust. Miss Maxine Tran is the Chief Investigator of the study.

How have patients and the public been involved in this study?

Ninety-nine patients and members of the public responded to an online questionnaire hosted by Kidney Cancer UK over a two-week period. Almost all of the responses were in favour and supportive of the general aims and concepts of the study. There are also two patient representatives on the study committee, who have helped in the drafting of this information sheet, consent form and study protocol. The patient representatives will also be attending the regular trial meetings to monitor the progress of the study. They will also be helping with disseminating the results of the study at patient education days, national conferences and online platforms.

Who has reviewed the study?

All research in the NHS is looked at by an independent group of people, called a Research Ethics Committee, to protect your interests. This study has been reviewed and given favourable opinion by East Midlands Research Ethics Committee. This study has also been reviewed by the Research for patient benefit programme panel for the National Institute for Health Research (NIHR) which is funding the study.

Contacts for further information

If you would like further information or have any questions about this study, please consult your consultant or contact us on the number below. You may also find it useful to contact cancer BACUP, an independent patient advisory group (Freephone 0800181199).

Mr David Cullen
Lead Nurse Renal cancer
07775687823

Miss Sara Hamilton
Lead Cancer Research Nurse
0207 794 0500
Sara.hamilton@nhs.net

Thank you for taking the time to read the information about the study.
