# Supplementary material for: Protocol for a feasibility study of a cohort embedded randomised controlled trial comparing NEphron Sparing Treatment (NEST) for small renal masses
Source: BMJ Open. 2019 Jun 11;9(6):e030965. doi: 10.1136/bmjopen-2019-030965 (PMC6577353; doi:10.1136/bmjopen-2019-030965)
Supplement: Reviewer comments [file bmjopen-2019-030965.reviewer_comments.pdf]

PEER REVIEW HISTORY

BMJ Open publishes all reviews undertaken for accepted manuscripts. Reviewers are asked to complete a checklist review form (**http://bmjopen.bmj.com/site/about/resources/checklist.pdf**) and are provided with free text boxes to elaborate on their assessment. These free text comments are reproduced below.

ARTICLE DETAILS

**TITLE (PROVISIONAL)**	Protocol for a feasibility study of a cohort embedded randomised controlled trial comparing NEphron Sparing Treatment (NEST) for small renal masses
**AUTHORS**	Neves, Joana; Cullen, David; Grant, Lee; Walkden, Miles; Bandula, Steve; Patki, Prasad; Barod, Ravi; Mumtaz, Faiz; Aitchison, Michael; Pizzo, Elena; Ranieri, Veronica; Williams, Norman; Wildgoose, William; Gurusamy, Kurinchi; Emberton, Mark; Bex, Axel; Tran, Maxine

This article was not externally reviewed at BMJ Open. Protocols that have been independently assessed prior to submission to BMJ Open are usually fast-tracked to publication on the grounds that further substantial changes will not be possible. This independent assessment will usually be external, independent review for both a substantial grant award from a non-commercial or government funder and ethics approval .
